# Addressing the Complex and Pressing Societal Issue of Air Pollution and Health

**DOI:** 10.5334/aogh.2672

**Published:** 2019-12-16

**Authors:** Jörg Hacker

**Affiliations:** 1 German National Academy of Sciences Leopoldina, DE

## Abstract

Air pollution is a pressing issue that needs to be addressed at all levels, more urgently than ever. Air pollution has been a recognized and serious health problem far longer than the beginning of industrialization. There are accounts from the Roman philosopher Seneca, who in a letter described some 2000 years ago how much better he felt after leaving “a ruinous mess of steam and soot”.

## Commentary

Air pollution is a pressing issue that needs to be addressed, more urgently than ever, at all levels. Air pollution has been a recognized and serious health problem far longer than the beginning of industrialization. There are accounts from the Roman philosopher Seneca, who in a letter some 2000 years ago described how much better he felt after leaving “a ruinous mess of steam and soot”.^1^

Successful attempts to control air pollution as well as resistance to any regulation has had a long tradition, too. One argument that has been brought up by opponents for more stringent air pollution control over and over again was that the scientific basis was insufficient. Opponents regularly called for more research before action could be taken.

This argument is no longer valid today. Today, we can paint an increasingly detailed picture of air pollution and its detrimental effects on human health: we know about the sources of air pollution, we know about the societal and economic contexts leading to air pollution, and most important, we know how air pollution affects the health of humans across the entire lifespan, across all systems and organs in the body, and across the natural world.

One characteristic of air pollution is that sources and effects can be far away from each other. Air pollutants can travel across borders, from the household to the street, but also several hundred or thousands of kilometers between countries. Hence, an internationally coordinated approach together with effective regional and local actions is needed.

Air pollution control and prevention involves all levels and sectors in society. It requires expertise from multiple scientific disciplines. The national academies of sciences and medicines are uniquely placed to address complex and pressing societal issues at the nexus of different policy areas. Academies of sciences are independent fora where the best scientists from all disciplines come together to present and exchange their findings and reflect upon solutions.

Because of this, the Academy of Science of South Africa, the Brazilian Academy of Sciences, the German National Academy of Sciences Leopoldina, the U.S. National Academy of Medicine, and the U.S. National Academy of Sciences have started the initiative on air pollution and health. The respective statement was released and handed over to the Brazilian, German, and U.S. permanent representative to the UN and to high-level UN representatives in June 2019. A small group of academies from different regions of the world began this initiative. We encourage other scientists, research organizations and further actors to pick the momentum and engage at levels where the impact is highest.

The German National Academy of Sciences Leopoldina strongly supports actions in the field of “Air Pollution and Health” at an international and national level. On request of Chancellor Angela Merkel, the Leopoldina made policy recommendations on air pollution and health in Germany this year. It is clear that the domestic agenda cannot be treated independently from international developments. It will therefore be important to bring the global problem of air pollution to the attention of a broad range of relevant stakeholders in politics and society. We encourage immediate action.

## Footnotes

Moral letters to Lucilius/Letter 104. (2019, May 8). In Wikisource. Retrieved 13:00, November 5, 2019, from https://en.wikisource.org/w/index.php?title=Moral_letters_to_Lucilius/Letter_104&oldid=9244901.

